# Supplementary material for: Immunogenicity and Protection from Receptor-Binding Domains of Toxins as Potential Vaccine Candidates for Clostridium difficile
Source: Vaccines (Basel). 2019 Nov 8;7(4):180. doi: 10.3390/vaccines7040180 (PMC6963439; doi:10.3390/vaccines7040180)
Supplement: Supplementary file 1 [file vaccines-07-00180-s001.pdf]

vaccines

MDPI

Supplementary Materials

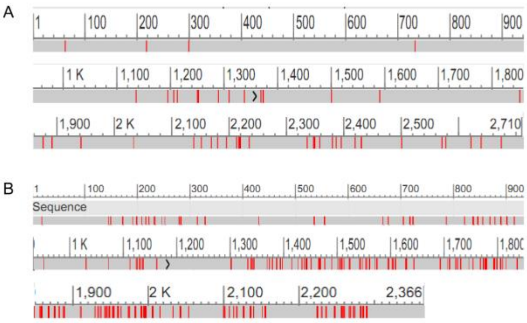

C

Toxin A identities (98.15%)			Toxin B identities (98.15%)		
Domain 1	Domain 2	Domain 3	Domain1	Domain 2	Domain 3
1-543	544-1832	1833-2710	1-544	545-1834	1835-2366
540/543	1275/1289	846/879	525/544	1186/1290	471/533
99.4%	98.9%	96.2%	96.5%	91.9%	88.4%

Figure S1. Blast results of toxin amino acids sequence in CD bacterium strain VPI10463 and strain ATCC BAA-1870. (**A**) Toxin A; (**B**) Toxin B; (**C**) The complete toxin A and toxin B. Red parts in A and B are different amino acids. Domain 3 is RBD of toxin.
